# Translational Challenges in Drug Therapy and Delivery Systems for Treating Chronic Lower Extremity Wounds

**DOI:** 10.3390/pharmaceutics16060750

**Published:** 2024-06-02

**Authors:** Danny Aljamal, Priya S. Iyengar, Tammy T. Nguyen

**Affiliations:** 1 Chan School of Medicine, University of Massachusetts, Worcester, MA 01655, USA; danny.aljamal@umassmed.edu (D.A.); priya.iyengar@umassmed.edu (P.S.I.); 2 Division of Vascular and Endovascular Surgery, Department of Surgery, University of Massachusetts, Worcester, MA 01655, USA; 3 Diabetes Center of Excellence, University of Massachusetts, Worcester, MA 01655, USA

**Keywords:** burn, chronic wound, diabetic foot ulcer, drug delivery systems, ischemic, venous

## Abstract

Despite several promising preclinical studies performed over the past two decades, there remains a paucity of market-approved drugs to treat chronic lower extremity wounds in humans. This translational gap challenges our understanding of human chronic lower extremity wounds and the design of wound treatments. Current targeted drug treatments and delivery systems for lower extremity wounds rely heavily on preclinical animal models meant to mimic human chronic wounds. However, there are several key differences between animal preclinical wound models and the human chronic wound microenvironment, which can impact the design of targeted drug treatments and delivery systems. To explore these differences, this review delves into recent new drug technologies and delivery systems designed to address the chronic wound microenvironment. It also highlights preclinical models used to test drug treatments specific for the wound microenvironments of lower extremity diabetic, venous, ischemic, and burn wounds. We further discuss key differences between preclinical wound models and human chronic wounds that may impact successful translational drug treatment design.

## 1. Introduction

**Figure 1:**
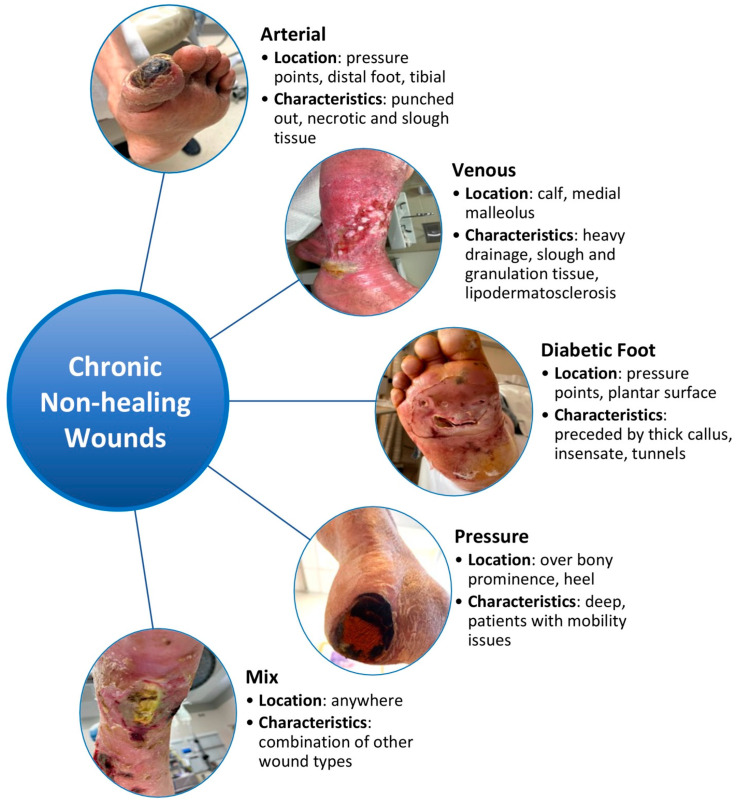
Types of chronic non-healing wounds by underlying etiology. The underlying etiology for chronic wounds can be identified based on location on the lower extremity and distinguishing wound characteristics [2].

Chronic non-healing wounds impact more than 6.5 million Americans and currently incur an annual healthcare cost of more than 25 billion dollars [1]. The prevalence and cost of chronic non-healing wounds are expected to increase with the rise of aging populations, diabetes, obesity, and cardiovascular disease. The etiology for a chronic non-healing wound may vary from arterial insufficiency, venous insufficiency, diabetes, pressure, and/or mixed presentations (Figure 1) [2]. Untreated lower extremity wounds can lead to severe infections, necessitating major lower extremity amputation for infection control and/or pain management. This process can significantly impact patients’ ability to work and move independently, especially for those with limited resources [3,4]. Treating lower extremity wounds poses several challenges, often involving multiple medical specialties and lasting up to 12 to 13 months on average, if successful [5,6,7]. Recurrence rates of lower extremity wounds affect up to 60% to 70% of patients, leading to functional loss and reduced quality of life [7,8,9]. Given these challenges and the increasing prevalence of non-healing lower extremity wounds, developing effective drug therapies is crucial.

Despite treating the underlying cause of a wound, chronic wounds may remain challenging to heal due to the hostile wound microenvironment. In humans, the chronic wound microenvironment is hypoxic with limited arterial perfusion, enriched with tissue destructive reactive oxygen species (ROS) and cytokines, and often harbors a thick layer of bacterial and/or fungal biofilm [10,11,12,13]. Initial wound injury disrupts local blood vessels, triggering a hypoxic microenvironment that activates hypoxia-inducible factor 1-alpha (HIF1α) to enhance angiogenesis mechanisms for restoration of tissue oxygen levels [14]. However, in chronic non-healing wounds, HIF1α signaling is impaired, leading to unresolved hypoxia [11,15,16]. Alongside hypoxia, chronic wounds remain in a pro-inflammatory state driven by phagocytic immune cells reliant on redox signaling and ROS production. Increase in ROS can result in cellular damage and a further reduction of local tissue oxygen availability [17]. This persistent hypoxia and compromised blood flow create a conducive environment for bacterial or fungal biofilm formation, which can further complicate the wound microenvironment [18,19].

The shared hostile wound microenvironmental factors often make it challenging for effective drug treatment, as evident by the few numbers of market-approved lower extremity wound drugs available. Currently, drug therapy and delivery system research on wound healing rely heavily on animal models, which may not accurately reflect the complexities of human chronic wounds. Animal models, though useful for early pre-clinical research, do not precisely mimic human physiology and diseases. This creates important challenges when trying to translate lab findings into successful treatments for humans. The translational gap between the human chronic wound microenvironment and the wound microenvironment found in animal models may therefore hinder effective drug treatment design for chronic wounds. To better understand how drug therapy and delivery systems can address the challenges of the human chronic wound microenvironment, this review will (1) outline the stages of normal wound healing and how it differs from the chronic wound microenvironment, (2) examine targeted drug therapies addressing hypoxia and angiogenesis, elevated ROS, inflammation, and the biofilm microenvironment of chronic wounds, (3) evaluate drug delivery systems applicable to chronic wounds, and (4) highlight research developments in drug treatments for diabetic, venous, ischemic, and burn wounds with an emphasis on human wound presentation compared to animal research models.

## 2. Stages of Wound Healing and Chronic Wound Microenvironment

**Figure 2:**
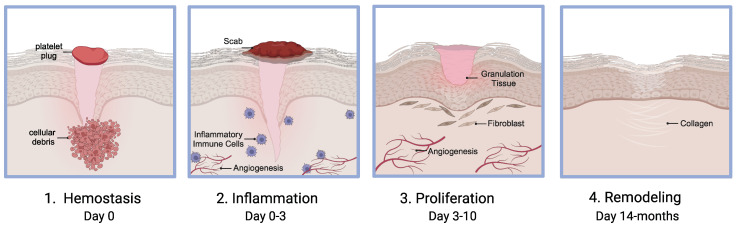
Stages of normal wound healing. The temporal four stages of normal wound healing progresses from hemostasis to inflammation, proliferation, and remodeling. The expected length of time in each wound healing stages is indicated. Image created in BioRender, agreement number HL26S82IJQ.

Wound healing requires an intricate synchronization of four different stages: hemostasis, inflammation, proliferation, and remodeling. Each stage is not mutually exclusive, but they often work in tandem to ensure adequate wound healing [2,20]. When one or more of the stages of wound healing fails to progress to the subsequent stages of healing, the wound becomes chronic (Figure 2).

### 2.1. Hemostasis

Injury to tissue causes disruption of blood vessels leading to extravasation of blood and its contents. The first step in wound healing involves hemostasis, which serves to stop the bleeding post injury. This involves constriction of the injured blood vessels along with activation of platelets that form a fibrin platelet plug. This serves as a cytokine-signaling source and temporary scaffold for infiltrating immune and fibroblast cells that are needed for wound healing [21,22,23,24]. The platelet plug and fibrin mesh also act as a barrier against harmful microorganisms.

### 2.2. Inflammation

Within the first 24 h, the inflammatory stage is in full effect, starting with an influx of leukocytes, including mastocytes, macrophages, fibroblasts, neutrophils, cytokines, and chemokines. Neutrophils are immediately recruited to the clot as they serve as the first line of defense against bacteria. Monocytes are recruited 48–96 h after injury and are activated to combat self and foreign antigens. These inflammatory cells release destructive lysosomal proteases, cytokines, and ROS to facilitate removal of cellular debris. With the ending of the inflammatory stage, angiogenesis, fibroblast migration and proliferation become more pronounced [12].

### 2.3. Proliferation

The proliferative phase is characterized by granulation tissue formation, contraction, and fibroplasia to establish a viable epithelial barrier and restore the vascular network via angiogenesis. This phase usually starts approximately 3–10 days after injury and normally takes days to weeks to complete. Molecular drivers for the proliferative phase include growth factors, such as transforming growth factor β (TGF-β), epidermal growth factor (EGF), fibroblast growth factor (FGF), platelet derived growth factor (PDGF), pro-angiogenesis factor, and vascular endothelial growth factor (VEGF) [2,9,12].

### 2.4. Remodeling

This last phase is considered the end point of wound healing. Remodeling begins approximately two to three weeks after injury and can last months to several years. This stage focuses on providing maximum tensile strength and recovery to normal tissue. During this phase, there is matrix deposition with degradation of type III collagen and increase in type I collagen. The collagen fibers become thicker and are placed in parallel to provide increased tensile strength [12]. The formation of mature type I collagen is critical; any disruptions in this process can result in delayed wound healing, potentially leading to the development of a chronic wound.

### 2.5. Chronic Wound Microenvironment

**Figure 3:**
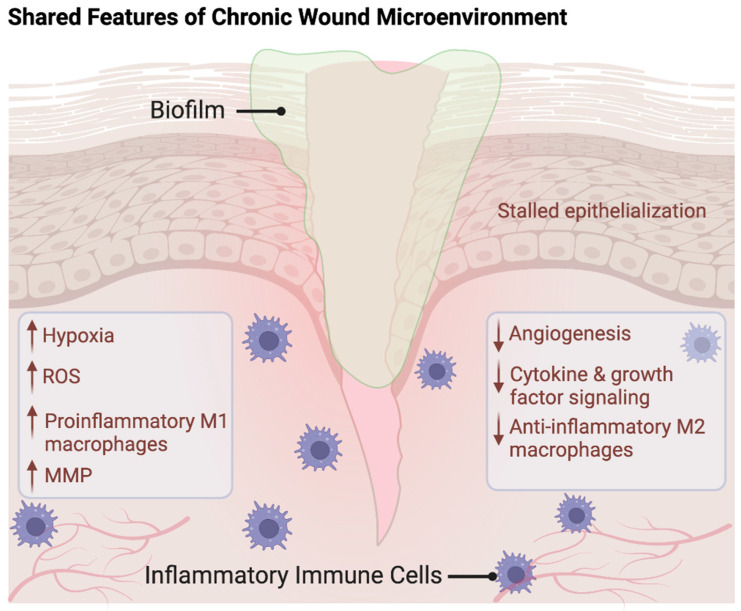
Common features of the chronic wound microenvironment. Wounds that fail to heal after 6–8 weeks are considered chronic. Chronic wounds may stem from different etiologies; however, they often have shared features that prevent progression towards healing. Chronic wounds often have a layer of biofilm that prevents epithelialization. Furthermore, the chronic wound microenvironment is hypoxic. The up arrow indicates an increase in ROS, inflammatory macrophages, and metalloproteases (MMP). The microenvironment can lead to a reduction in angiogenesis, cytokine, and growth factor signaling, and reduced anti-inflammatory macrophage recruitment as indicated by the down arrow. Image created in BioRender, agreement number ZU26R8×4PV.

Unlike normal wound healing, chronic wounds fail to proceed through the four stages of wound healing. It is unclear if there is a specific mechanistic determinant that prevents the progression of normal wound healing stages and pushes a wound to become chronic. Most chronic lower extremity wounds occur from an inciting traumatic insult that initiates the hemostasis stage, similar to normal wound healing [2,6,7,8,12]. However, unlike normal wound healing, several studies have demonstrated that chronic wounds, independent of their etiology, often fail to transition from the inflammatory stage to the appropriate proliferation and remodeling wound healing stages [9,12,20]. In a study comparing single cell RNA velocity trajectory analysis of human wound tissue from healing diabetic foot ulcers to chronic non-healing diabetic foot ulcers, chronic non-healing wounds demonstrated decreased cell differentiation velocity trajectory towards fibroblasts, smooth muscle cells, and macrophages. This suggests that diabetic chronic wounds are less likely to be in the proliferative wound healing stage compared to healing wounds [25]. The failure of chronic wounds to enter the proliferative stage has been linked to hypoxia, ROS, inflammatory cytokines, proteases, and dense biofilm (Figure 3) [9]. Below, we outline recent research on drug therapies designed to address these shared characteristics found in the chronic wound microenvironment.

## 3. Targeted Drug Therapy Designed for the Chronic Wound Microenvironment

### 3.1. Hypoxia and Angiogenesis

The chronic wound microenvironment is reported to be hypoxic and result in impaired angiogenesis, leading to poor wound healing (Figure 1). Under normal conditions, tissue hypoxia stimulates the expression of VEGF by stabilizing HIF1α, which in turn promotes wound angiogenesis. However, in chronic wounds, HIF1α becomes destabilized, resulting in a loss of VEGF expression and the inhibition of angiogenesis irrespective of tissue hypoxia. This interruption in angiogenesis can hinder progression to the proliferative stage of healing [16,26,27,28]. To address the reduction of angiogenesis in the chronic wound microenvironment, several drug therapies targeting the HIF1α and VEGF pathways have been developed; however, they yield mixed results in wound healing between animal models and human studies [29,30,31,32].

Under non-pathological hypoxic conditions, HIF1α is necessary for the expression of multiple angiogenic growth factors [33], cell motility [34], and recruitment of endothelial progenitor cells during wound healing [14,35]. In a normal setting, hypoxia leads to HIF1α stabilization by oxygen-dependent hydroxylases. In contrast, chronic non-healing diabetic foot ulcer biopsies demonstrate reduced HIF1α expression, suggesting that hyperglycemia is sufficient to impair the response to hypoxia [15]. Based on these data, drug therapies aimed to promote HIF1α stabilization are under active investigation. Currently, there are two commonly used drugs for HIF1α stabilization. The first drug is a 2-oxoglutarate analogue dimethylxalylglycine (DMOG), an iron chelator, and the second drug is a reactive oxidant scavenger deferoxamine (DFX). Both drugs are hydroxylase inhibitors that stabilize and activate HIF1α to promote angiogenesis in wounds of animal models [10,11,32,36]. In diabetic mouse models, 2 mM DMOG and 1 mM DFX topical application on wounds can stabilize HIF1α levels, enhancing both the downstream transcription of genes critical for angiogenesis and the wound healing under normoxic hyperglycemia conditions [10]. Comparative analysis between 1 mM DMOG and 1 mM DFX in diabetic murine wound models demonstrate enhanced wound healing with topical DFX over DMOG. Interestingly, there was no discernible difference in wound closure effectiveness between DMOG and DFX treatments in 21-month-old murine wound models. These findings indicate that while HIF1α stabilization can improve wound healing in both diabetic and age-related wounds, the unique ROS scavenger properties of DFX specifically enhance healing in diabetic wounds [37]. In humans, DFX is an injectable iron chelator that has been Food and Drug Administration (FDA) approved since 1968 to treat acute or chronic iron toxicity, such as in hemochromatosis [38]. There have been several off-label research uses of topical and injectable application of DFX for chronic wound treatment using in vitro human endothelial cells [36], murine models [36,39,40,41,42,43,44,45], and chicken [46] over the past decade. However, no formal clinical trial has been performed. There is active interest in achieving a new drug application (NDA) to expand the clinical indication of DXF for wound healing; however, federal clinical trial approval will depend on more large-animal studies [38].

Alternative strategies to enhance angiogenesis in chronic wounds include usage of VEGF through viral transduction, mRNA transfection, and engineered fibroblast overexpression in animal wound models [29,30,31]; however, results have been less convincing in human trials [29,47]. In addition to HIF1α and VEGF, hypoxia can result in impaired angiogenic chemokine stromal cell derived factor-1 (SDF-1) expression in chronic wound tissue. Overexpression of SDF-1 in a hyperoxia setting have synergistically shown an increase in neovascularization and wound healing in murine diabetic wounds [48,49], but this has not been demonstrated in humans [50].

### 3.2. Inflammation

Several types of chronic wounds are often reported to be stalled in the pro-inflammatory phase based on the observed abundance of inflammatory cytokines and ROS secretion by immune cells within the wound microenvironment (Figure 1) [9,28]. It is therefore thought that by reducing the pro-inflammatory signaling within the wound microenvironment, chronic wounds will be able to transition into the proliferative phase and progress to healing. Based on this hypothesis, strategies for drug therapies to reduce the pro-inflammatory innate immune response either directly targeting cytokine signaling or ROS have demonstrated promising results in several animal models.

#### 3.2.1. Reactive Oxygen Species Scavenger

Chronic wounds are enriched in ROS, and several ROS scavengers and antioxidants have demonstrated promising results in wound healing [51]. Off-label use of DXF, an iron chelator that is thought to be a ROS scavenger, has been shown to promote HIF-1α stabilization and improve wound healing [10,11]. Other antioxidant drugs, such as edaravone, captopril, and cerium dioxide have also demonstrated accelerated wound healing in several animal models [52,53,54]. Edavarone and captopril are market-approved as a ROS scavenger for cerebral ischemia and hypertension, respectively; however, their dose-dependent therapeutic role in wound healing has yet to be translated to human studies [52,53].

#### 3.2.2. Inflammatory Cytokines

Directly mitigating the pro-inflammatory phase in the chronic wound microenvironment is also an attractive therapy target to promote wound healing. Several studies have reported improvement in diabetic wound healing with drugs that dampen the pro-inflammatory cytokines identified within the wound microenvironment. Reducing levels of interleukin-1 and interleukin-17 [55,56] and promotion of M2 anti-inflammatory macrophage using konjac glucomannan-modified SiO_2_ nanoparticles have independently demonstrated wound healing and global anti-inflammatory effects [57]. Others have reported that introduction of interleukin-8 into diabetic wounds stimulates the recruitment of mesenchymal stem cells and endothelial cells for neovascularization [58,59]. These preclinical studies have prompted interest in human clinical trials for drug therapies that augment the cytokine immune profile within a chronic wound microenvironment. In a multinational clinical trial, ON101, a topical cream composed of *Plectranthus tiamboinicus* and *Centella asiatica* plant extracts, has been shown to reduce inflammatory M1 wound macrophages by suppressing the inflammasome pathways interleukin-1 and interleukin-6 while activating anti-inflammatory M2 macrophages through fibroblast proliferation and migration (Table 1). The results thus far have been promising, demonstrating complete healing in 60.7% of participants over a 16-week period compared to 35.1% of those receiving an absorbent dressing of sodium carboxymethylcellulose hydrocolloid fiber (Hydrofiber; ConvaTec Ltd., Paddington, UK) [60].

#### 3.2.3. Inflammatory Byproducts

The development of chronic wounds often leads to a buildup of inflammatory byproducts such as fibrin, leukocytes, microorganism biofilm, dead cells and proteins that develop into fibrinous and/or eschar tissue overlying the wound bed and subsequently inhibits granulation tissue formation [18,19,61]. The mechanism by which fibrinous/eschar tissue inhibits granulation tissue formation is unclear; however, debridement of the fibrinous/eschar tissue either by sharp excisional removal or topically with drug targeted therapy improves granulation tissue formation. Topical drug therapy for enzymatic or autolytic debridement of fibrinous/eschar tissue includes a variety of market-approved agents ranging from recombinant collagenase or proteolytic enzymes to a mixture of balsam peru, castor oil, and trypsin [2,62,63].

**Table 1:** Current human clinical trials for chronic non-healing wounds. There are several clinical trials approved in the United States and internationally for non-healing wounds that vary from diabetic wounds, peripheral arterial disease, and burns. Current drugs for chronic wounds vary in drug target mechanism and drug delivery type.

Target Mechanism	Drug Delivery/Type	Drug	Clinical Trial	Wound Type	Active Component(s)	References
Biofilm	Topical Agent	TP-102	Phase 2b, US, India (NCT05948592)	Diabetic Wounds	- Cocktail comprised of five lytic bacteriophages against *Staphylococcus aureus*, *Pseudomonas aeruginosa*, and *Acinetobacter baumannii*.	[64]
Inflammatory Stage	Hydrogel	ON101	Phase 3, US (NCT04962139)	Diabetic Wounds	- PA-F4: Attenuates M1 macrophages - S1: Activates M2 macrophages	[65]
Inflammatory Stage	Topical Agent	EscharEx	Phase 2 completed, US (NCT04817228). Ready for Phase 3	Diabetic Wounds	- Debridement agent: Concentrate of proteolytic enzymes enriched in bromelain	[62]
Inflammatory Stage	Topical Agent	NexoBrid	Phase 3 completed, US (NCT02148705). Pending FDA approval	Burn Wounds	- Debridement agent: Concentrate of proteolytic enzymes enriched in bromelain	[66]
Inflammatory Stage	Topical Agent	NexoBrid	Phase 3, US (NCT02278718) Pending FDA approval	Burn Wounds	- Debridement agent: Concentrate of proteolytic enzymes enriched in bromelain	[66]
Inflammatory Stage	Topical Agent	ILP100	Phase 2, Sweden (NCT05608187)	Diabetic Wounds	- Genetically modified *Limosilactobacillus reuteri* R2LC expressing CXCL12-α	[67]
Inflammatory Stage/Biofilm	Topical Agent	AMP PL-5	Phase 3, US, (NCT06189638)	Diabetic Wounds	- Membrane active antimicrobial peptide against both gram-negative and gram-positive bacteria	[68]
Inflammatory Stage/Growth Factors	Cell Engineering	AUP1602-C	Phase 2, Germany, Italy, Poland (NCT06111183)	Diabetic Wounds	- Genetically modified *Lactococcus cremoris* expressing FGF-2, IL-4, and CSF-1	[69]
Inflammatory Stage/ROS	Intravenous	Plasma Activated Normal Saline	Phase: Not Applicable. China (NCT05924867)	Miscellaneous	- Rich in reactive oxygen groups (ROS) and reactive nitrogen groups (RNS)	[70]
Inflammatory Stage/Tissue Regeneration	Topical Agent	ENERGI-F703	Phase 3, US, Taiwan (NCT05930210)	Diabetic Wounds	- AMPK agonist	[71]
Tissue Regeneration	Hydrogel	Timolol	Phase 3, US (NCT03282981)	Diabetic Wounds	- Beta-adrenergic receptor agonist	[72]
Tissue Regeneration	Dressing	PLCL/Fg	Phase 4, China (NCT06014437)	Diabetic Wounds	- Nanofibrous poly(L-lactide-co-caprolactone) with formulated porcine fibrinogen	[73]
Tissue Regeneration/Stem Cell	Topical Agent	TTAX01	Phase 3, US (NCT04450693)	Diabetic Wounds	- Cryopreserved human umbilical cord product	[74]
Tissue Regeneration/Stem Cell	Silver Scaffold	Umbilical cord mesenchymal stem cells	Phase 2, China (NCT05319106)	Venous LE ulcer wounds	- Umbilical cord mesenchymal stem cells	[75]
Tissue Regeneration/Stem Cell	Topical Agent	Umbilical cord mononuclear cells	Phase 3, China (NCT04689425)	Diabetic Wounds	- Umbilical cord mononuclear cells	[76]
Tissue Regeneration/Stem Cell	Hydrogel	ALLO-ASC-DFU	Phase 3, Korea (NCT04569409)	Diabetic Wounds	- Allogeneic adipose stem cells	[77]
Tissue Regeneration/Stem Cell	Hydrogel	ALLO-ASC-SHEET	Phase 2, US (NCT03754465)	Diabetic Wounds	- Allogeneic adipose stem cells	[78]
Tissue Regeneration/Stem Cell	Hydrogel	ALLO-ASC-SHEET	Phase 2, US (NCT04497805)	Diabetic Wounds	- Allogeneic adipose stem cells	[78]

### 3.3. Micro RNA

Several human venous chronic wounds have reported an association with either increased or decreased levels of micro-RNA (miR) when compared to normal wound tissue [79,80]. These observations have led to more mechanistic work using animal wound models to suggest a role for aberrant miR expression at all stages of wound healing [81]. Overexpression of several different miR, such as miR-26a, miR-200, miR-31, miR-21, miR-34, miR-424, miR-516, and miR-132, has been demonstrated to reduce fibroblast proliferation and migration in vitro and in animal wound models, therefore suggesting that the increased level of miR within the chronic wound microenvironment may impair the transition from the inflammatory to proliferative wound healing phases [79,80,82,83,84]. In fact, the use of targeted drug therapy for specific miR has been shown to improve wound healing in murine diabetic wounds [85,86,87].

### 3.4. Growth Factors

The chronic wound microenvironment is often reported to be depleted of critical growth factors that are essential for the proliferative phase [88]. Currently, the only market that approved recombinant human growth factor for wound healing in Europe and the United States is PDGF [89]. However, challenges with protein drug stability and controlled release have resulted in safety concerns, particularly regarding its use in patients with neoplastic co-morbidities [90]. Overall, the application of exogenous growth factors provides a promising treatment for chronic wounds, and there are ongoing clinical trials, such as for VM202, a human hepatocyte growth factor [91]. However, developing systems for sustained localized drug delivery to mitigate off target effects and increase efficacy may be challenging.

### 3.5. Biofilm and Wound Infection

The hostile environment created by pro-inflammatory cytokines, elevated matrix metalloproteases, increased neutrophils, hypoxia, and reduced growth factors is an ideal ground for microbial growth [61]. This bacterial colonization of the wound bed, often by pathogens that are resistant to antibiotics, is called biofilm, and it propagates persistent inflammation, preventing wound healing [18]. Biofilms are present in over 60% of biopsy specimens from chronic wounds and 6% of biopsy specimens from acute wounds (Figure 1) [18]. Biofilm colonization is often poly-microbial, with *Staphylococcus aureus* (*S. aureus*), *Pseudomonas aeruginosa* (*P. aeruginosa*), *Candida albicans*, and beta-hemolytic streptococci as the primary causes of delayed wound healing and infection [92]. Interestingly, colonization by the community of pathogens varies by depth, where anaerobic bacteria tend to settle deeper than aerobic bacteria [93]. While *S. aureus* has been described to colonize the upper layers of the chronic wound bed, *P. aeruginosa* is found in the deeper layers [92]. Newly formed biofilm is most susceptible to therapeutic interventions in a window of 48–96 h before maturation. Mechanical debridement of biofilm can be performed surgically or through a bedside procedure [2,61]. While debridement significantly removes the biofilm, the remaining free bacteria can rapidly reform, leading to the need for frequent debridement. Each new formation of biofilm has its own specific characteristics in terms of pathogen makeup and mutation development, which can make treatment specificity challenging to develop. Debridement along with PCR identification of specific pathogens [61] can allow for a more targeted treatment approach. Furthermore, next-generation RNA sequencing may provide a more comprehensive identification of the biofilm transcriptome to guide treatment specificity; however, this technology’s cost is currently prohibitive in a clinical setting. Sodium hypochlorite and boric acid, hypochlorous acid, or iodine-based products are more cost effective for treating *P. aeruginosa* infections [61,94]. Soft tissue or localized wound infection is more amenable to antibiotic treatment. Although topical antibiotics have not been shown to clinically improve chronic wound infection, oral antibiotics are efficacious at treating more diffuse soft tissue infections associated with chronic wounds. Alternatively, the use of antimicrobial nanovesicles can facilitate the controlled release of drugs only in the presence of pathogens to mitigate off-target effects [95]. Silver- and copper-based drug nanoparticles have also been shown to be efficacious at preventing bacterial biofilm development through a bactericidal mechanism [96,97]. Given how biofilm is embedded in an extracellular polymeric matrix (EPM) produced by itself, silver ions in wound care dressing for infected chronic wounds may impede biofilm formation by competing with the EPM for binding sites. It is likely that a multifaceted approach involving multiple agents simultaneously is best to facilitate healing and tackle the stubborn biofilm [61,93].

### 3.6. Proteases

Multiple different cell types within the wound microenvironment secrete matrix metalloproteases (MMPs) in response to cytokine and growth factor signaling. Overexpression of MMPs in the chronic wound microenvironment can lead to disruption of the extracellular matrix (ECM), leading to impaired granulation tissue formation and epithelialization required for the remodeling phase of wound healing [98]. Several studies have targeted the inhibition of ECM metalloproteases in chronic wounds through gene therapy, such as small interfering RNA (siRNA) [99]. For example, the application of siMMP-9 to diabetic wounds in mice inhibits the translation of MMP-9 by promoting mRNA degradation. Application of siMMP-9 to chronic murine wounds can increase collagen deposition and wound healing [100]. However, several human clinical trials with MMP inhibitors have been discontinued due to development of a musculoskeletal syndrome from off-target drug effects. Currently, the only market-approved MMP inhibitor is for periodontitis [101].

## 4. Drug Delivery Systems for Chronic Wounds

**Figure 4:**
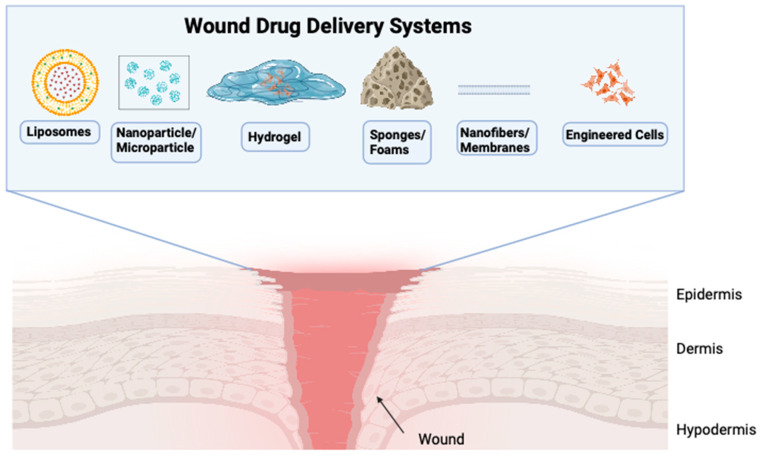
Chronic Lower Extremity Wound Drug Delivery Systems. Common wound drug delivery systems used for chronic lower extremity wounds. Image created in BioRender, agreement number BQ26U95IML.

Developing a drug delivery system that will provide sustained drug concentration to a chronic wound microenvironment that is plagued with proteases, alkaline pH, and biofilm is imperative for effective treatment. It is important to optimize the chronic wound bed to mitigate the hostile microenvironment prior to drug delivery. Wound bed optimization can reduce excessive protease secretion, provide pH balance, and control biofilm through the TIME (tissue, infection/inflammation, moisture balance and the edge of the wound) guidelines [2,63]. In addition, the drug delivery system must be compatible with the physical and chemical properties of the drug therapeutic agent. For example, small molecule drugs, gene therapy, siRNA, growth factors, cytokines, and cells (biologics) will each vary in requirements to maintain drug activity. Therefore, effective treatment will rely on pairing a drug with the appropriate delivery system. To address the biocompatibility, biodegradability, and antigenicity requirements for effective drug delivery, the current drug delivery systems are reviewed below (Figure 4, Table 2).

### 4.1. Liposomes

The use of liposomes or lipid-based carriers allow for an encapsulated drug with high affinity to cell membranes while passing through biological barriers. Liposomes are a self-assembled amphipathic phospholipid bilayer with a hydrophobic surface that allows for cellular entry through endocytosis while maintaining a hydrophilic aqueous core that is compatible for encapsulating the targeted drug. Liposomal-based drug delivery systems can be covalently linked to polyethylene glycol (PEG) or through non-PEG technology that enables reduced immunogenicity and antigenicity. Liposomes are a versatile drug delivery system because of the wide range of sizes available, from 20–1000 nm, and diverse membrane lamellarity options to accommodate sustained release and increased bioavailability. Currently, there are no market-approved liposomal drugs delivery systems to treat wounds; however, there are several liposome-based market-approved drugs for various cancers, viral vaccines, photodynamic fungal diseases, photo enhancement for ocular diseases, and analgesics [104]. In animal models for chronic wounds, there have been promising preclinical results for wound healing using liposomal-based delivery of recombinant SDF-1, insulin-like growth factor 1 (IGF-1), fibroblast growth factor (FGF), and miR-21 and simvastatin [117,118,119,120]. The versatility of liposomes to accommodate a wide range of drug size and their low immunogenic properties make them an attractive drug delivery system for wound microenvironments. Liposomes can readily be delivered topically or through subcutaneous injections directly to the wound. The local drug application can mitigate unwanted off-target effects and increase drug efficacy. The disadvantages of liposomes are cost, leakage, fusion, solubility, and short half-life destabilization with low pH that may be present in chronic wound microenvironments (Table 2).

### 4.2. Nanoparticles

Nanoparticles (NPs) refer to a wide range of inorganic, polymer, or lipid-based materials that range from 1–100 nm. Drug therapy can be encapsulated by nanoparticles and designed for cell- or tissue-specific drug delivery [108]. Nanoparticles can also be loaded onto hydrogels for acute or chronic wound healing. Like liposomes, nanoparticles are also versatile in their size and can be designed to cell/tissue specificity. However, unlike liposomes, nanoparticles can readily enter lymphatic systems and lead to off-target effects beyond the intended wound treatment (Table 2).

One of the most common inorganic-based NPs utilize metals. Silver, gold, and zinc NPs are often utilized in wound care dressings due to their antibacterial properties and low toxicity. Silver nanoparticles (AgNPs) have broad spectrum activity against bacteria and fungi have been used in chronic wound dressings like silver-anticoat and polyvinyl alcohol nanofibers. Combined with antibiotics like amoxicillin, clindamycin, penicillin, and vancomycin, AgNPs can increase their antibacterial effect and have the potential to combat multidrug resistant infections [96,121]. However, it is important to be mindful that long-term usage of antibiotics in general can lead to drug-resistant strains of pathogens [93,121].

In addition to metal-based NPs, there are a variety of polymer-based NPs made of synthetic (e.g., poly-caprolactone), natural (e.g., gelatin or chitosan), nonbiodegradable materials (e.g., cyanoacrylate or poly(lactic-co-glycolic acid), or biodegradable materials (e.g., polyurethane). Polymer-based NPs are advantageous over other nanocarriers because they are easy to synthesize, cost-effective, biocompatible, non-immunogenic, and have some biodegradable and water-soluble options. Cationic polymers form more stable complexes that facilitate cellular migration more efficiently than cationic lipid-based NPs. There are several NP-based drugs approved by the FDA; among these, 29% were polymeric-NPs, 22% were liposomal, and 21% were lipid-based [122].

Niosomes are non-ionic lipid-based NPs that can encapsulate hydrophobic or hydrophilic drugs. These lipid-based NPs were used in cosmetics since the 1970s, but have now gained much attention as a drug delivery system for their ability to retain the biodegradability, versatility, and biocompatibility features of traditional nanoparticles while maintaining a low toxicity profile, storage stability, and low-production cost [123]. The lipid bilayer of niosomes allows for enhanced targeted drug delivery to cells, similar to liposomes; however, unlike liposomes, this drug delivery system manufacturing process does not require expensive phospholipids. The noisome synthesis process is like the methods available for liposomes with the primary exception of incorporating non-ionic surfactants instead of relying on phospholipids. Niosome vesicle size can be adjusted using techniques similar to liposome preparation, like sonication, extrusion, mechanical, sonocavitation (ultrasound), or homogenization.

The potential use of a niosome delivery system to encapsulate drugs targeted at wound healing is highly appealing due to its versatility and lower production cost than liposomes [124]. Recent studies have explored the use of niosomes encapsulating substances like methylene blue [125], antioxidants, or atorvastatin [126] in murine wound healing models. However, these studies have neither shown significant improvement in wound healing rates in animal models nor have they been used in human chronic wounds.

### 4.3. Microparticles

Like nanoparticle properties, microparticles (MPs) can vary in their material composition of either metal, polymer, lipid, liposomes, or carbon-based materials that range from 1–1000 μm in size. An advantage of the larger size microparticles is that the size prevents entry into the lymphatic system or the blood-brain barrier, therefore mitigating some of the off-target effects that may be observed with NPs [108] (Table 2).

### 4.4. Scaffolds

Scaffolds provide a foundation for cell migration, adhesion, and tissue remodeling, while allowing for wound drainage and angiogenesis. There are several different types of scaffolds for wound treatment in the form of hydrogels, sponges, foams, nanofibers, films, or membranes.

#### 4.4.1. Hydrogels

Hydrogels are composed of either insoluble or soluble polymers that can be engineered to bind to active drugs through chemical or physical bonds. The hydrogel drug delivery system can be delivered through a variety of methods, such as topically as a gel or patch, injection, surgical implantation, or orally (Table 2). Hydrogels have low immune reactivity and can provide spatial and temporal controlled release of therapeutic agents to provide optimal treatment efficacy. The bioavailability and biodegradable properties of hydrogels depend on the type of polymers used and the crosslinking method. Hydrogels can be made of either natural polymers (such as chitosan, alginate, cyclodextrin, and collagen) or synthetic polymers (such as *N*-isopropylacrylamide (NIPAM), acrylamide, polyvinyl alcohol (PVA), and PEG) that are either chemically or physically crosslinked [111,127]. The versatility of hydrogels allows for applications ranging from a vehicle to elicit an adaptive immune response to promote wound healing [128]; a drug delivery system such as VEGF plasmids [129], interleukin-8 [59], or Edavarone [52]; cell-printing onto endothelial cells expressing VEGF [130,131]; or FGF [132] into diabetic murine and pig wounds. Currently, the only FDA-approved drug for diabetic foot ulcers is a hydrogel-based delivery for human recombinant PDGF-BB (Regranex^TM^/becaplermin gel, Ortho-McNeil Pharmaceutical, Raritan, NJ, USA).

Hydrogels can be engineered to physically swell or shrink in response to various microenvironment stimuli, such as pH, pressure, organic compound concentration, the presence of specific biomolecules, and redox state. The physical ability of responsive hydrogels to change shape in order to facilitate active drug release in the presence of a microenvironmental stimulus makes them attractive as a drug delivery system for a chronic wound microenvironment. Reactive hydrogels engineered to respond to temperature and redox levels can deliver antioxidants and encapsulated SDF-1 into diabetic wounds of murine models [49]. Currently on the market, Regan Lab offers a topical autologous platelet-rich plasma hydrogel to treat venous, pressure, and diabetic ulcers in humans [133]. Additionally, clinical trials using hydrogel forms of the beta-adrenergic receptor antagonist—timolol and esmolol—have been tested to treat chronic venous ulcers [134,135,136,137]. In addition to being a drug delivery system, hydrogels can also provide hydration to chronic wounds that are desiccated. However, hydrogels may not be an ideal drug delivery system for high drainage output wounds, since they have limited absorption capacity, and the high drainage may pose a physical barrier for drug delivery (Table 2).

#### 4.4.2. Sponges and Foams

Sponges and foams are made of porous materials to provide a three-dimensional (3D) framework structure synthesized from a variety of materials, including natural organic materials (e.g., agar, chitosan, alginate, RNA, and DNA) or synthetic organic polymeric complexes (e.g., PVA, polylactic acid, and polyactic-coglycolic acid (PLGA)). Sponges typically have a denser and more solid structure, while foams have a higher proportion of gas bubbles within their matrix. Sponge pore sizes can range from micro-scale (1–1000 μm) to nano-scale (<1000 nm) and encapsulate either hydrophilic or hydrophobic drugs, whereas foams can be a solid or liquid material that is microporous (pore diameter below 2 nm), mesoporous (pore diameter 2–50 nm), and macroporous (pore diameter above 50 nm) [112,113]. The pore sizes can be a limitation on the capacity to load and retain drugs, especially those with large molecular sizes or specific chemical properties (Table 2).

Microsponges have been incorporated into lotions, creams, and other topical formulations for treating skin conditions such as acne, hyperpigmentation, and wounds. They have a high storage capacity, elasticity, capillary action, and controlled drug release capabilities. Foams can provide a larger surface area for drug release, potentially leading to faster drug release rates compared to sponges. Sponges and foams have a high surface-to-volume ratio created by numerous pores, allowing for available inner space with more absorption sites on both external and internal surface areas to facilitate drug shielding capacity. The exceptional absorbent nature of sponges has heightened their appeal as hemostatic wound products. A recent study utilized a micro-channel alkylated chitosan sponge on hemorrhagic perforated liver wounds in rats and pigs. This sponge exhibited improved hemostasis, resistance against bacterial growth, promoted proliferation of liver fibroblasts, and enhanced tissue vascularization [138]. However, despite their dual function of delivering active drugs and acting as wound dressings, sponges and foams may face limitations due to their high water absorption capacity, which could impact pore size and subsequently decrease the rate of drug delivery (Table 2) [112,139].

Microsponges have recently been studied for their potential to serve as scaffolds for cell encapsulation. The rigid support of microsponges can facilitate cell attachment and provide a conducive environment for cellular interactions, mimicking physiological conditions and promoting cell survival. Specifically, collagen-based microsponges have been extensively used to culture various cell types, such as vascular endothelial cells and hepatocytes. This type of application allows for higher efficiency in cell seeding and faster cell growth compared to conventional scaffolds [112]. Additionally, the use of cells encapsulated on a microsponge scaffold with hydrogels enriched in antibacterial agents have also been shown to provide the dual function of a biodegradable wound dressing with delivery of therapeutic treatment agents [140,141,142].

The synthesis of sponges or foams for drug delivery depends on the physicochemical properties of the encapsulated drug molecules and scaffold polymers. Sponges are typically synthesized using commonly employed methods such as double-emulsion solvent evaporation, quasi-emulsion solvent diffusion, and liquid–liquid suspension polymerization. Furthermore, several novel preparation technologies, such as lyophilization, electrohydrodynamic atomization, and self-assembly have been developed to streamline processing and enhance production reproducibility [112]. Foams, which are typically produced by churning and expanding gas within a properly formulated continuous phase, are commonly employed. The resulting foams can vary in characteristics based on factors such as the composition of the continuous phase and the specific process parameters utilized [113].

#### 4.4.3. Nanofibers

Nanofibers offer a unique drug delivery system characterized by their small diameter distribution, high porosity, gas permeability, and high surface area design to resemble tissue ECM (Table 2). These structural properties can support cell proliferation and migration that is crucial for wound healing. Nanofibers derived from either poly (α-esters) (PLA, PGA, and PLGA), chitosan, gelatin, HA, or alginate [143] can be coated with drugs, metal, chemicals, or cells for wound treatment [144]. Their porous network allows for efficient oxygen diffusion, moisture absorption, low toxicity, and high drug delivery efficacy to the wound site. Furthermore, the moisture retaining properties of nanofibers also make them ideal non-adherent wound dressings that create a moist wound microenvironment beneficial for dry chronic wounds.

Electrospinning is the most common method to process polymer nanofibers [143]. Electrospinning fibers is a scalable process and requires a high voltage power supply, a container of polymer solution, a pump, and a collector. There are several different types of electrospinning techniques available to accommodate various drug and cell properties for optimizing drug encapsulation, cell seeding, release rate, and drug stability. Electrospinning is widely used due to its cost-effectiveness and efficiency in producing uniform nanofibers with diameters ranging from 5 to 100 nanometers, significantly smaller than fibers produced by other methods [20,145]. By providing a porous, non-occlusive scaffold, electrospun nanofibers can be layered for prolonged drug release. In a recent study, a multilayer polylactic acid nanofiber matrix was loaded with three drugs, phenytoin, sildenafil citrate, and simvastatin, each in a separate layer to target a different wound healing phase. The multilayer nanofiber patch demonstrated different drug release rates in a diabetic rat wound model [114]. This drug delivery system can thus facilitate nutrient and gas exchange as well as adsorption of exudate, act as a barrier against bacterial contamination, and serve as a drug delivery system to the wound site [20,146].

#### 4.4.4. Films and Membranes

Effective drug delivery to a wound is dependent on the physical characteristics of the wound microenvironment. This has led to significant interest in integrating wound dressing properties with drug delivery systems. For instance, if a wound is dry, drug absorption can be challenging; therefore, hydrogels or nanofibers can provide both hydration and drug delivery. On the other hand, wounds with high exudate drainage pose difficulties in making drug delivery systems contact the wound tissue. In such cases, sponges and foams are used to absorb excess drainage, although their high-water uptake can limit their effectiveness as drug delivery systems (Table 2).

To meet the dual needs of effective drug delivery and wound dressing, films and membranes present a unique solution. These scaffolds can be loaded with drugs or pre-seeded with autologous or allograft cells and embedded in a thin film or sheet-like material. Films and membrane consist of natural and/or synthetic polymers or a blend that can be manufactured using various techniques such as self-assembly, phase separation, and electrospinning. Films are thin, transparent biopolymer layers that are biocompatible and biodegradable, making them suitable for delivering drugs to wounds [147]. Their thin and flexible nature allows for wound shape conformability as well as the exchange of oxygen, water vapor, and carbon dioxide. While their ability to absorb exudate makes them ideal for shallow or minimal-draining wounds, one of the most appealing features of films is their transparency. This allows healthcare providers to monitor the wound’s healing progress without having to remove the dressing or disrupt drug delivery. In a recent study, a sodium alginate-based hydrocolloid film was used to deliver vicenin-2, a plant derived compound, to wounds in a diabetic rat model. The study demonstrated that 50 uM of vicenin-2 coated film was sufficient to accelerate diabetic wound healing, reduce inflammatory markers, and enhance fibroblast proliferation within the wound microenvironment when compared to film alone and allatonin-coated film [148]. These data demonstrate the effective use of film in potentially altering the wound microenvironment with drug delivery while acting as an occlusive wound dressing.

For wounds with moderate drainage levels, membranes are preferable to films. Membranes have the capacity to absorb excess exudate while maintaining a balanced moisture environment that is conducive to wound healing. Membranes can effectively deliver drugs to wounds under drainage pressure and minimize disruption to the wound bed and drug delivery system by requiring less frequent dressing changes. For example, in a recent study, a mesh-like electrospun membrane delivered atorvastatin and bone marrow-derived mesenchymal stem cells (MSCs) to rat wound models to demonstrate the effective use of membranes as an engineered skin substitute drug delivery system [149]. However, films and membranes have limitations in absorbing large volumes of wound exudates, making them unsuitable for highly exuding wounds; for these circumstances, the use of a foam or sponge delivery system may be preferred (Table 2).

### 4.5. Cell Engineering

The emergence of genetically modified cells or bacteriophages has provided an additional modality for a targeted drug delivery system to treat chronic wounds. There are several clinical trials using genetically modified bacteriophages to target antibiotic-resistant bacteria in chronic wounds that can lead to biofilm formation (Table 1) [150,151]. In addition to bacteriophages, non-pathogenic bacteria have also been used as a drug delivery system. For example, AUP-16 is a genetically modified *Lactococcus cremoris* non-pathogenic bacterium that expresses human basic fibroblast growth factor (FGF2, bFGF). Interleukin-4 (IL4) and macrophage colony stimulating factor (CSF1, mCSF) is topically applied to non-healing diabetic ulcers, providing a consistent supply of cytokines and growth factors that have been shown to promote wound progression into the proliferative and remodeling healing stages. Results from the AUP-16 Phase 1 clinical trial reports complete wound healing in 83% of patients, a much higher healing rate compared to other treatment modalities thus far (Table 1) [152]. However, excitement for this drug delivery system should be cautioned, as there is potential for off-target effects and unintended bacterium colonization.

Stem cell technology is a growing field of interest in wound treatment since these cells can be autologous, provide localized cytokine and growth factors, and subsequent differentiation into epidermal and dermal cells to replace the damaged skin. Mesenchymal stem/progenitor cells (MSC) can be autologously harvested from the bone marrow, peripheral blood, adipose tissue, epidermis, or umbilical cord blood, and subsequently genetically modified for therapeutic use [153,154,155]. Induced pluripotent stem cells (iPSCs) are derived from somatic cells and can serve as an alternative autologous stem cell source. The autologous, non-immunogenic properties and local paracrine immune modulation potential of stem cells make them an attractive target for a drug delivery system. For example, bone-marrow-derived MSC that is transduced to overexpress CXC motif chemokine receptor 6 (CXCR6) can enhance in vivo localization to the wound microenvironment and increase neovascularization, MMP-2, and re-epithelialization in diabetic wounds [156]. However, the pro-inflammatory and hostile wound microenvironment causes difficulties regarding its stem cell viability. Improvement in stem cell recruitment and viability within the wound microenvironment is an ongoing research topic that has ranged from genetic modification to enhance homing to the wound, or the use of a 3D spheroid patch delivery system to provide a more protective environment for stem cells [157].

## 5. Advances in Drug Therapy for Chronic Leg Wounds

### 5.1. Diabetic

Patients with diabetes mellitus (DM) have a 30-fold increased risk of developing diabetic foot ulcers (DFUs), with 43% requiring lower extremity amputations [8]. Currently, the sole hydrogel-based FDA-approved drug for DFU treatment is human recombinant PDGF-BB, known as Regranex^TM^ or becaplermin gel (Ortho-McNeil Pharmaceutical in Raritan, NJ, USA). Given the high prevalence of DFUs, the risk for a major lower extremity amputation, and the limited treatment options available, numerous clinical trials (Table 1) and translational research have been dedicated to advancing DFU healing strategies.

Recent advancements in high-throughput analysis of human DFUs have shed light on potential mechanisms that may promote healing. When comparing patients whose ulcers heal (DFU-Healers) and those who do not heal (DFU-Non-healers) using single cell sequencing and spatial transcriptomics, DFU-Non-healers were found to exhibit a higher prevalence of cytotoxic natural killer T cells, indicating a shift in T-cell subpopulations, along with specific fibroblast subpopulations showing overexpression of MMP-1, MMP-3, MMP-11, HIF1A, CHI3L1, and TNFAIP6, as well as increased M1 macrophage polarization [25]. These differentially expressed gene differences between DFU-Non-healers and DFU-Healers corroborate findings from previous research animal models. However, it is crucial to note that many of the variations observed between human DFU-Healers and DFU-Non-healers pertain to immune cell functions. Over the past decade, research has elucidated significant differences in immune cell types, cytokine signaling, and transcriptomic profiles between humans and corresponding murine wound healing research models [158,159,160,161]. Thus, murine models commonly used for wound healing investigations should be used cautiously when drawing conclusions about immune responses to specific drug therapies.

To bridge the gap between murine animal model wounds and human DFUs, researchers have employed splinted murine wounds with an added bacterial biofilm to mimic human diabetic wounds, which are often colonized with bacterial biofilm. In a recent study, murine diabetic wounds inoculated with methicillin-resistant *S. aureus* (MRSA) or a carbapenem-resistant *P. aeruginosa* biofilm were treated with a hydrogel-based dressing containing antibacterial cationic polyimidazolium and antioxidative N-acetylcysteine to enhance wound healing [162]. Another investigation using murine diabetic wound models with biofilm demonstrated that a microneedle bandage delivering dopamine-coated hybrid nanoparticles containing selenium and chlorin e6 (SeC@PA) promoted wound healing by reducing reactive oxygen and nitrogen species within the wound microenvironment [13].

Despite potential limitations, murine models remain a valuable resource for testing drug treatments for DFUs due to their small size and ease of care, making them a cost-effective in vivo research model. Recent studies have utilized murine diabetic wound models to evaluate cell-engineering drug therapies and drug delivery systems. For example, engineered adipose stem cells (ASCs) modified to continuously express hepatocyte growth factor (HGF) and CXCL12 were administered to diabetic mouse wounds using a hydrogel delivery system, resulting in improved healing rates compared to control groups. These engineered ASCs were delivered using lipid nanoparticles to carry self-amplifying RNAs (saRNAs), which encode the genes of interest and viral replicase, allowing for sustained expression of desired proteins at lower doses [163]. Another approach involves introducing exogenous cells, such as human umbilical vein endothelial cells engineered to overexpress VEGF-165 through a lentiviral vector, encapsulated in an all-peptide hydrogel and applied to diabetic rat wound models [130]. Alternatively, microorganisms have also been introduced into murine mouse models. For example, Haematococcus (HEA), a microorganism that naturally produces the antioxidant Astaxanthin (AST), was delivered to murine diabetic wounds using a conformable hydrogel delivery system. The release of oxygen and AST antioxidants from HEA to the wound under light stimulation was found to enhance wound healing outcomes in murine mouse models [164].

Aside from introducing engineer cells to DFUs, gene silencing therapy using micro-RNA (miR) was also explored for wound healing in murine diabetic wounds. Injection of miR-221-3p into these wounds demonstrated enhanced healing by regulating signaling pathways in keratinocytes [165].

Despite significant innovations in drug therapy and delivery systems for DFUs in preclinical studies, as highlighted above and over the past decade, many promising interventions often fail to translate into successful human clinical trials. This underscores the urgent need for preclinical models that better reflect human DFUs to advance effective treatments. One possibility for the lack of translatability of current animal wound models to successful human clinical trials may stem from key differences between the human immune system and animal models. The use of humanized mouse models in cancer therapy, such as an immunocompromised nude mouse host reconstituted with human immune cells, has revealed new understandings of immune cell infiltration into tumors that was not previously shown using a murine immune-system-based model [166]. Given these significant immune system differences, it may be advantageous to use humanized mouse models for future wound healing experiments.

### 5.2. Venous

Despite accounting for 70–80% of all lower extremity ulcers and impacting 1.5–3% of the general population, there is less abundant research on venous ulcers than DFUs. Animal models for venous ulcers commonly use mice overloaded with iron-dextran via intraperitoneal or subcutaneous injection until there are skin hemosiderin deposits [167]. Skin hemosiderin discoloration is only one of several features of human venous ulcers, and the described animals therefore may not reflect other features of human venous wounds that are relevant to wound healing such as venous valvular insufficiency, varicose veins, and leg swelling. Given that there is a paucity of animal models for venous wounds, most research studies conducted on venous ulcers have been in humans. The current standard of care for venous ulcers has historically focused on wound care dressing. Furthermore, venous ablation procedures paired with compression wound care dressing results in an over 80% wound healing rate over 2 years [168]. Currently, there are two FDA drugs based on human fibroblast cells cultured on a bioabsorbable polymer scaffold approved for venous ulcer treatment: Dermagraf^TM^ (Organogenesis in Canton, MA, USA) and Apligraft^TM^ (Organogenesis in Canton, MA, USA). Apligraft^TM^ is also indicated for treatment of DFUs. Additionally, off-label use of pentoxifylline, indicated for vascular claudication, has been shown to accelerate venous ulcer wound healing in small case studies [169]. Over the past two decades, there has been a paucity of new drug treatments tested for venous ulcers. Currently, there is a Phase 2 clinical trial in China testing the use of mesenchymal stem cells on venous ulcers (Table 1).

### 5.3. Ischemic

Peripheral arterial disease (PAD) affects approximately 7% of the population in the United States, with an estimated 1.3% experiencing chronic limb-threatening ischemia, a severe form of PAD that can lead to ischemic ulcers. Ischemic ulcers are particularly concerning, as they can progress to gangrene and ultimately require amputation [170]. The initial treatment of ischemic ulcers involves surgical or endovascular revascularization. However, these conventional revascularization methods are limited by anatomical constraints, relying on the presence of large and medium-sized arteries for repair.

As a result, there is growing interest in drug therapies aimed at promoting angiogenesis and arteriogenesis by enhancing collateral artery formation and neovascularization. Currently, there are no FDA-approved drugs or active clinical trials specific for treating ischemic ulcers (Table 1). There have been previous attempts to intravenously or intramuscularly introduce gene therapy to ischemic ulcers by enhancing VEGF expression; however, phase II/III clinical trials yielded variable results [171,172]. In a recent study, researchers utilized cells from the adipose stromal vascular fraction to seed a clinical-grade skin substitute. This engineered skin substitute was then applied to wounds in an ischemic hindlimb mouse model, resulting in the development of a mature vascular network comprising arteries, capillaries, veins, and lymphatic vessels [173].

### 5.4. Burn

Deep burns are chronic wounds that penetrate the skin’s layers to affect blood vessels, nerves, and hair follicles, causing severe pain and prolonged healing time. Additionally, deep burn wounds are highly susceptible to multidrug-resistant infections, often necessitating treatment targeting bacterial biofilm and necrotic tissue formation. Currently a topical enzymatic drug to debride necrotic tissue from burn wounds is in Phase 3, pending FDA approval (Table 1).

Several animal models are used to simulate full-thickness burn wounds, including direct and radiant heat applications to the shaved skin of mouse, rat, pig, and rabbit models. These full-thickness burn wounds pose an elevated risk of thermal dysregulation and increased water loss due to the loss of the epidermal and dermal skin layers. Therefore, hydrogels are considered excellent drug delivery systems as they enhance water retention in the wound environment.

Most in vivo burn wound research is conducted in murine models. For instance, a recent study incorporated a nanocomposite of zinc oxide (ZnO) and Ag NPs (with antibacterial properties) mixed with vitamin A and E into wheat gluten films, which were applied to murine burn wounds. This composite film improved burn wound healing rates [145]. Alternatively, hydrogel-encapsulated adipose-derived stem cells can be applied to murine burn wound models for enhanced wound healing and neovascularization [174]. Although these studies demonstrate improvements in wound healing, it is debatable whether these models are translatable to human burn wounds since human wounds often are complicated with severe biofilms that are not present in these animal studies.

To address the impact of biofilm and infection in burn wounds, several recent studies have tested different drugs using a murine burn model inoculated with bacteria. Recently, avian-generated IgY antibodies against two strains of *P. aeruginosa* were sufficient to reduce biofilm formation and increase bacteria hydrophobicity, although their impact on wound healing remains unclear. It is important to note that IgY antibodies were injected into murine burn wounds concurrently with bacterial infection, followed by re-dosing at 2-h, 24-h, 48-h, and 72-h intervals post-infection, and therefore may not be as efficacious if only administered post infection [175].

Conducting burn research using animal models can be challenging due to wound heterogeneity. To address this challenge, a research group recently developed a chorioallantoic membrane assay (CAM) as a surrogate model for animal experiments. In this model, burn wounds were induced on the CAM using surgical cautery, allowing observation of post-burn angiogenesis. This model holds promise for more scalable drug discovery efforts [176].

## 6. Discussion and Conclusions

There have been notable advancements in targeted drug therapy and drug delivery systems for chronic lower extremity wounds over the past two decades, showing promising results in preclinical studies and setting the stage for human clinical trials. However, despite these efforts, several clinical trials focusing on chronic lower extremity wounds have failed to yield FDA-approved therapies over the past two decades. This discrepancy between preclinical success and clinical trial outcomes raises questions about the underlying knowledge gap.

Both targeted drug therapy design and drug delivery systems rely heavily on preclinical animal models for successful human wound healing treatment development. Animal models, while valuable for initial research, do not perfectly mirror human physiology and pathologies, leading to unique challenges in translating findings from a targeted drug therapy and drug delivery system perspective. Effective drug therapy treatment will rely on using preclinical models that accurately reflect the human non-healing wound pathophysiology, anatomy, and cellular make up. In this regard, the most commonly used animal models for preclinical wound healing studies are murine and porcine models. Pigs resemble humans in anatomy and immune function, but their size brings logistical challenges such as increased cost and housing requirements [177]. Mice are therefore a preferred wound healing model organism due to their size, ease of handling, genetic manipulability, and short reproductive cycle, rendering them a cost-effective model. However, mice and humans have notable differences in skin anatomy that are important to consider when designing targeted drug therapies. In mice, the hypodermis lies above the panniculus carnosus, a thin layer of striated muscle that is closely attached to the skin and fascia that results in wound healing through the contraction of the panniculus carnosus [178], whereas human wounds heal through re-epithelialization. To address this difference in wound healing between mice and human, splint devices can be sutured around full-thickness excisions to prevent contraction and induce healing by epithelization [178]. However, splinted wound models only address the anatomical differences between human and murine wounds.

At a cellular level, there are likely important differences between preclinical animal wound models and human chronic wounds that may have hindered the success of clinical trials. Several preclinical studies have shown promising results by targeting hypoxia pathways via HIF-1α activation, enhanced VEGF levels [10,11,29,30,31,32], ROS scavengers [10,11,52,53,54], and SDF-1 overexpression in hyperoxia conditions [48,49]. However, when similar drug targeted approaches are tested in human clinical trials, such as intravenous or intramuscular VEGF enhancement for ischemic ulcers, the therapeutic outcomes are less convincing in phase II/III trials [171,172]. This may suggest that there are other critical cellular and molecular factors in human wounds that have not yet been tested in preclinical animal models. Since the wound healing process relies on key immune cells for cytokine signaling to promote cellular proliferation (Section 2.2), the differences in immune cell proportions and function between human and murine models may impact wound healing outcomes. Murine and human immune cell difference can impact immune surveillance, antigen presentation, and cell communication [158,159,160,161]. To bridge these immune-cell-mediated differences that may impact targeted drug therapy design, humanized mice may offer a unique approach. A humanized mouse model utilizes immunocompromised nude mice engrafted with human stem cells to create an in vivo human immune system model that can be leveraged to study how human immune cells impact wound healing. In a recent study investigating the human acute immune response to MRSA wound infection, a humanized mouse model underwent a human skin transplant procedure prior to acute MRSA skin inoculation [179]. Wound tissue biopsy showed the presence of human monocytes and macrophages in both the host mouse blood and grafted human skin tissue post-MRSA skin graft inoculation. This indicates the model’s capability to mount a human-like immune response to cutaneous wounding [179]. Alternatively, to simulate human wounds more accurately in preclinical studies, several groups have utilized an ex vivo human skin wound model to test for wound healing drug therapies [162,175]. Ex vivo human skin wound models can provide valuable insights into critical cell types for wound healing in human skin that may not be present in animal models. These advances in developing more human-like wound healing preclinical models for targeted drug design are promising and may provide better translational success in human clinical trials.

In addition to the importance of preclinical wound models that accurately reflect human wound healing physiology for targeted drug therapy design, preclinical models that represent the human chronic wound microenvironment challenges to drug delivery are also needed. Since preclinical animal wound models are often sterile, and human chronic wounds arise from heterogenous etiologies complicated by biofilm, the translatability in wound-healing drug research can be hindered. Thus, addressing the hostile wound microenvironment with strategies targeting destructive proteases [99,100] and biofilm [95,96,97] may provide better insights for preclinical drug delivery system development. For example, leveraging reactive hydrogels to respond to various microenvironment stimuli, such as pH, proteases, pressure, organic compound concentration, the presence of specific biomolecules, and redox state may provide improved drug delivery and prevent drug degradation. The physical ability of responsive hydrogels to change shape to facilitate active drug release in the presence of a microenvironmental stimulus makes them attractive as a drug delivery system for a chronic wound microenvironment. For instance, murine diabetic wounds with a bacterial biofilm were treated with a hydrogel-based dressing containing antibacterial cationic polyimidazolium and antioxidative N-acetylcysteine to show enhanced wound healing [162]. It will be of interest to see if the results from the murine diabetic wound with biofilm model will translate into human studies.

Advancing effective drug delivery systems for clinical use has not only been largely limited by the paucity of animal wound models that accurately reflect human wounds, but also due to the high cost of production and drug delivery system designs that have poor bioavailability and unwanted off-target effects. For example, liposomes offer benefits such as increased bioavailability and reduced drug degradation by encapsulating drugs. The use of liposomes has shown promise in animal studies for delivering drugs like SDF-1, IGF-1, FGF, and miR-21 [117,118,119,120]. However, they may face logistical challenges like high costs and potential leakages. On the other hand, hydrogels are versatile scaffolds currently used to encapsulate human recombinant PDGF-BB (sold as Regranex^TM^) for treating DFUs. However, the cost of this hydrogel-based drug remains substantial, exceeding $1000 for a 15-g dose of Regranex^TM^.

Aside from cost, a major limitation of hydrogel-based drug delivery is their limited absorption capacity, resulting in decreased drug delivery efficiency in high drainage output wounds. Designing a drug delivery system that can both deliver active drugs and act as wound dressing is an appealing approach to overcome barriers in the wound microenvironment. There is a clear unmet need to improve drug delivery design to suit the wound microenvironment and offer a scalable, cost-effective solution for drug manufacturing.

## 7. Future Directions

Chronic wounds are a significant burden on healthcare resources, affecting approximately 1–2% of the global population [1]. Despite the large demand for wound healing drug treatments, there has been a discrepancy between preclinical success and clinical trial outcomes. This translational gap raises questions about the validity of current preclinical models for drug design and research. Wound drug design and delivery systems have traditionally relied on sterile preclinical animal models that differ from human wound anatomy, pathophysiology, immune cell profile, and chronic wound microenvironment. These differences may hinder the translational outcomes in targeted drug therapy design and delivery systems to human chronic wounds. More recent progress in developing complex humanized mouse models that closely mimic the human immune system in wound healing, incorporating ex vivo human skin platforms, and integrating biofilm into current small animal wound models may provide clinically relevant models for future research on targeted wound healing drug design and drug delivery systems.

## Figures and Tables

**Table 2:** Drug delivery systems used for chronic lower extremity wounds. Common drug delivery systems used for lower extremity chronic wounds are listed with the current drug application modality, advantages, and disadvantages of each system.

Drug System	Drug Application	Advantages	Limitations
Liposomes	- Topical - Transdermal - Injection	- Versatile size option (20–1000 nm) - Specific cell or tissue targeting - Encapsulate hydrophilic and hydrophobic drugs - Biodegradable - Low antigenicity - Low toxicity	- High production cost - Short half-life - Variable drug kinetics - Limited drug loading capacity - Leakage and fusion - Low solubility - Poor stability [102,103,104,105,106]
Nanoparticles	- Topical - Transdermal - Injection	- Versatile size option (1–1000 nm) - Modifiable for cell/tissue specificity - Encapsulate hydrophilic and hydrophobic drugs - Flexible composition - Biodegradable - Enhance stability - Improve drug solubility - Controlled drug release	- High production cost - Short half-life - Off-target effects (enters lymphatic/brain) - Limited drug loading capacity [107,108]
Microparticles	- Topical - Transdermal - Injection	- Versatile size option (1–1000 µm) - Specific cell or tissue targeting - Skin retention - Flexible composition - Biodegradable - Does not enter lymphatic or cross blood brain barrier	- High production cost - Short half-life [109]
Hydrogel	- Topical - Transdermal - Injection - Oral - Surgical implant	- Low antigenicity - Controlled release - Biodegradable - Reactive to stimuli - High water content good for dry wounds - Versatile application	- Limited wound absorption capacity - Limited drug loading capacity - Swelling can change drug release - High production cost - Restricted shelf life [110,111]
Sponges/Foams	- Topical - Transdermal - Oral - Inhalation - Intraperitoneal	- Function as wound dressing - High drug loading capacity - Sustained release - Versatile pore size - Biocompatibility	- Limited specificity of drug release - Infectious - Restricted shelf-life - Swelling can change drug release [112,113]
Nanofiber/Membranes	- Topical	- Low antigenicity - Biodegradable - Efficient drug loading - Controlled drug release - Can deliver wide range of drug type - Modified for cell/tissue specificity	- High-cost production - Prone to aggregation - Can have limited mechanical strength [20,114,115]
Engineered Cells	- Topical - Transdermal	- Specific cell or tissue targeting - Continuous drug production - Biocompatible - Regenerative potential	- High safety regulation - Unpredictable lifespan - Immunogenic - Off target effects [116]
